# Anteversion of the Uterus of Four Years Standing—Reduction

**Published:** 1874-12

**Authors:** J. H. Lowe

**Affiliations:** Atlanta, Ga.


### ANTE-VERSION OF THE UTERUS OF FOUR YEARS STANDING REDUCED WITH A UTERINE SOUND.

By J. H. LOWE, M.D., of Atlanta, Ga.

During the last year I was called to see Mrs. ——, 39 years of age, whom I found suffering from an attack of remittent fever, with rather more nervous symptoms than are usual in such attacks, as great restlessness, indisposition to sleep, and great despondency. The fever readily yielded to the usual remedies—mercury, quinine and opium—and in a few days she was convalescent. Before taking my leave of her, she told me she wished to consult me in regard to another trouble, from which she had suffered for the past four years. She called my attention to the ginger-bread colored spots upon her face, and the peculiar appearance of her finger-nails, and dead appearance of her hair, stating that for four years she had been a great sufferer. Upon inquiry I found that she was the mother of four children, the youngest five years old. For the past four years she had menstruated very imperfectly, not longer than one day at any one time. Had profuse lucorrhœa, and more or less pain and uneasiness all the time. The peculiar appearance of the hair and nails, with the ginger-bread spots, had existed since the commencement of her ill health. She informed me that she had taken almost everything, had been treated by four physicians, but felt that she had gradually grown worse under their treatment. I suggested that she had some disease of the womb, with perhaps some displacement, and that, to determine its character, it would be necessary for me to make a vaginal examination, both digital and with the speculum. She said she had never been examined, and very much disliked to submit to such an examination; but after impressing her with the necessity of such a procedure, she reluctantly consented.

As soon as she had entirely recovered from her attack of fever I examined her while in the dorsal position, and was unable to detect the os uteri, either with the finger or by means of the cylindrical speculum, the only instrument I had with me. On my next visit I used Sims' speculum, with patient on her elbows and knees, with a good light, and found, high up in the hollow of the sacrum, the os uteri, it being the most marked case of ante-version that I had ever seen. The question then presented itself, what was to be done? How could I best replace the organ in its normal position?

Having nothing better with me, I bent a silver sound to an angle of about one hundred and twenty-five degrees, and succeeded in hooking it in the os. I found, however, great difficulty in bringing it down with Sims' speculum in place, and the patient in the elbow and knee position. I then removed the speculum, and placed her in the lateral position, still retaining the bent sound in the womb. I now introduced the cylindrical speculum, passing the sound through the speculum as it was introduced. After several efforts while in this position, I finally succeeded in bringing down the os to its normal position, thus completely reducing the ante-version. To my great gratification, I found that after holding it in position for some time, that the os did not recede. I found upon the wall, near the os, an ulcerated surface the size of a dime, which readily yielded to a few applications of a solution of the nitrate of silver.

She was now put upon the volatile tincture of guaiac in drachm doses, three times a day, and a pill composed of aloes, myrrh and soap. At the first menstrual flow after adjusting the womb she menstruated three days; and the second, which came on at its regular time, continued four days, and was pretty healthy in quantity and appearance. In a few months she was entirely relieved, the hair and nails regaining their normal appearance, and the ginger-bread colored splotches on her face entirely disappeared.

